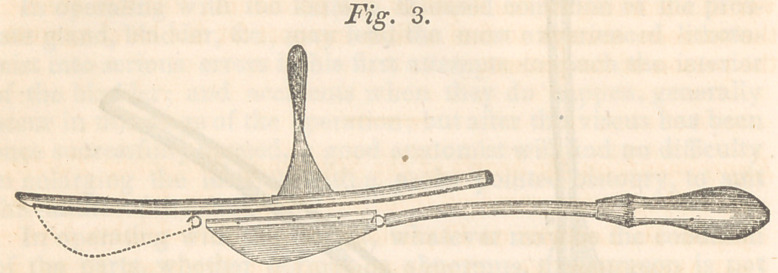# Contributions to General Surgery

**Published:** 1845-07

**Authors:** W. H. Elliot

**Affiliations:** Fellow of the American Society of Dental Surgeons. Plattsburgh, N. Y.


MEDICAL EXAMINER,

AND

RECORD OF MEDICAL SCIENCE.

NEW SERIES—No. VII.—JULY, 1845.

### ORIGINAL COMMUNICATIONS.

*Contributions to General Surgery.* By W. H. Elliot, D.D.S, Fellow of the American Society of Dental Surgeons. Plattsburgh, N. Y.

### THE CUTTING GORGET.

Since the days of Ammonius, no instrument has been invented for the operation of lithotomy which has been used with such general success, and, at the same time, liable to so fatal accidents, as the cutting gorget. The principles upon which this instrument acts is correct; yet there are few lithotomists in this country who have sufficient practice to enable them to gain a mastery over its defects; but when by a long experience the requisite skill is acquired by the surgeon, in plunging this instrument, to do it without allowing it to be disengaged from the staff, or to wound the fundus of the bladder, it cannot be denied that it is vastly superior to any other instrument ever used; this is abundantly proven in the unprecedented success of Professor Dudley. The smooth, straight forward incision made into the bladder by the cutting gorget, affords a much safer passage for the urine while it is disposed to flow through the wound, than the irregular and indirect opening produced by the knife.

It has been urged as a reason why the gorget should not be used, that it does not in all cases make an opening into the bladder sufficiently large to facilitate the egress of the calculus. We may infer from this, that it would be better to make the incision as large as the safety of the parts will allow, without previously ascertaining the size and figure of the stone, further than can be done by the introduction of the sound. But would it not be more in accordance with the correct principles of surgery, to first learn the exact size, shape and condition of the stone, and then enlarge the incision according to the necessities of the case, rather than endanger the final success of the operation, by making the incision unnecessarily large.

In operating with the knife, a diseased condition of the prostate gland, bladder, &c., may lead the most experienced lithotomist into serious errors in his first attempts to reach the interior of the bladder; and accidents when they do happen, generally occur in this stage of the operation; but after this viscus has been once successfully opened, a good anatomist will find no difficulty in enlarging the incision with a probe pointed bistoury, to suit the nature of the case.

In operating with the gorget, whatever may be the condition of the parts, whether normal or abnormus, the surgeon is not liable to be led astray; and the principle accident he can anticipate from the use of this instrument, is its slipping from the groove of the staff, and its being driven too high, too low, or entirely through the bladder. Then, if by any means we can so connect the staff and gorget that no accident whatever can separate them, or prevent them from doing their several offices in the most perfect manner, we shall have produced an instrument which may be used, to say the least, by the *less* experienced lithotomist with more safety than any other. To this end we have made some slight attempts, the result of which may be seen in the following cuts: [*See next page.*

Fig. 1 is a simple staff and gorget. The staff is hollow and open upon one side from the point where the external incision reaches it through the membranous portion of the urethra to the inferior end. The shaft of the gorget is split down for the reception of the blade, and has a small bulb upon the end of it. The dotted lines at A show the shape of the narrow end of the blade which confines it in its place, while a small screw through the bulb and blade at B secures the broad end. The opening in the staff is widened considerably at C for the reception of the bulb, which when pushed a little toward the inferior end of the staff, cannot be disengaged from it, the opening being too narrow for the bulb to pass out at any place but at C.

The mode of operating with this instrument is the same as that of the common gorget, except that the gorget is withdrawn as soon as plunged, and the forceps directed by the staff or index finger.

*Fig.* 1.

Fig. 2 is intended to represent the short staff and gorget. The staff is slightly curved and hollow, with an opening upon one

*Fig.* 2.

side of its convex surface reaching its whole length, having a handle about three inches in length standing at right angles with the body of the staff, and fastened upon its concave side. The shaft of the gorget is accurately fitted to the inside of the staff, so as to pass lightly through it from end to end, is split down at one end for the reception of a thin steel blade, and at the other end for a handle, both of which project out through the opening in the staff while in use, as is shown by the dotted lines.

*Fig.* 3.

Fig. 3 is a modification of fig. 2; the staff is precisely the same in every particular. The blade of the gorget is directed entirely by the staff, it being connected to the shaft and handle only by a loose joint; it has a back fastened upon it, which is fitted to the inside of the staff, and passes through it, as does the shaft of the gorget in fig. 2. The back of the blade only passes into the staff. The joint between the blade and shaft prevents any rubbing or catching of one part upon another, in case any considerable lateral force should be given to the handle of the gorget, and any awkwardness or irregularity of motion in the hand that gives the force, can have no influence whatever upon the hand that guides it.

The method of operating with the short staff and gorget is as follows: After a free external incision has been made, and the membranous portion of the urethra opened, the operator holding the upper end of the long staff or sound with his left hand, while with his right he introduces the short staff through the incision along the groove of the long staff into the bladder, the long staff may then be withdrawn. The operator then seizes the handle of the short staff, holding it perpendicularly between the thumb and fingers of his left hand, resting the back of his fingers firmly against the anterior perineum, at the same time pressing the body of the staff tight against the arch of the pubes; then with his right hand he introduces the gorget into the staff and pushes it forward to the bottom of the first incision, the lips of which being held apart by an assistant. The gorget then being secure from all accidents, may be plunged into the bladder with considerable rapidity, and instantly withdrawn. The index finger of the right hand may then be introduced, guided by the staff to feel for the stone; if the incision prove large enough for the calculus to pass out without lacerating the parts, the forceps may be passed in, guided by the staff and the staff removed; if the incision prove too small, a probe pointed bistoury passed in upon the finger is the most suitable instrument for enlarging it.

It would be well to have the handle of the short staff moveable, so that the distance between it and the end of the staff may be varied according to the depth of the perineum; this will enable the surgeon always to find a firm support for his hand against the person of the patient.

## Figures and Tables

**Fig. 1. f1:**
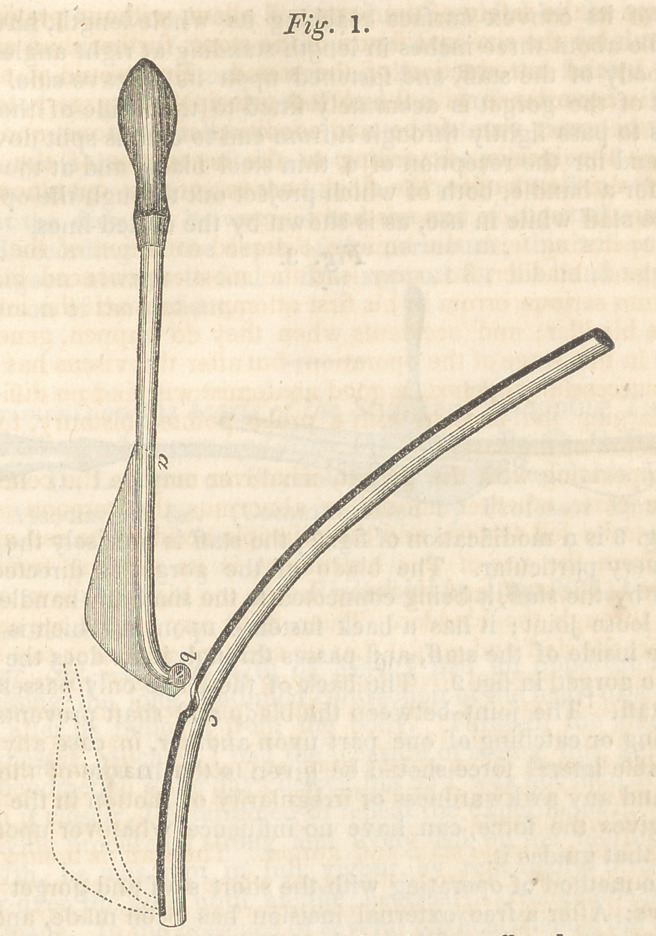


**Fig. 2. f2:**
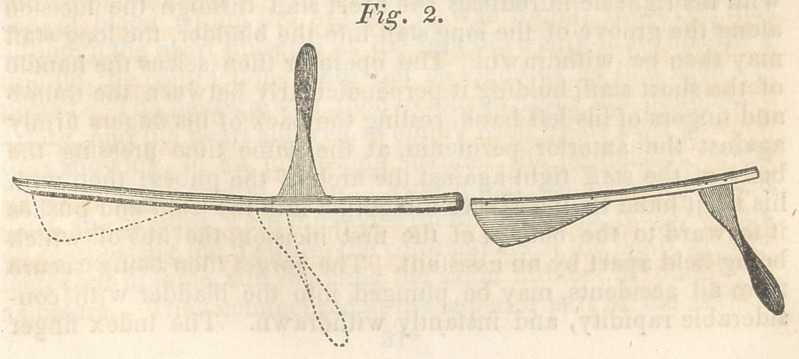


**Fig. 3. f3:**